# Knowledge, attitudes and practice of communities of Wolaita, Southern Ethiopia about long-lasting insecticidal nets and evaluation of net fabric integrity and insecticidal activity

**DOI:** 10.1186/s13071-016-1494-5

**Published:** 2016-04-22

**Authors:** Zewdneh Tomass, Bereket Alemayehu, Meshesha Balkew, Dawit Leja

**Affiliations:** Department of Biology, Biomedical Science Division, Wolaita Sodo University, College of Natural and Computational Sciences, P.O.Box 138, Wolaita Sodo, Ethiopia; Addis Ababa University, Aklilu Lemma Institute of Pathobiology (ALIPB), P.O. Box 1176, Addis Ababa, Ethiopia

**Keywords:** Malaria prevention, Long-lasting insecticidal nets, Fabric integrity, Insecticidal activity, Wolaita community, Southern Ethiopia

## Abstract

**Background:**

Optimal use of long-lasting insecticidal (LLINs) for malaria prevention depends on mass distribution, the users’ perception and behaviour of local malaria vectors. This study was aimed at assessing knowledge, attitude and practice (KAP) of communities about LLINs and fabric integrities and insecticidal activities of nets under use in Wolaita zone, Sothern Ethiopia.

**Methods:**

Semi-structured interview questionnaires were used to collect data on KAP variables and WHO cone bioassay was used to test the insecticidal activity of sampled nets against an insectary colony of *Anopheles arabiensis*. Holes and repairs on surfaces of sample nets were counted and categorized following WHO guidelines to assess their fabric integrities. Chi-square (χ^2^) tests were used to verify associations between the demographic profiles of the respondents and their responses to KAP questionnaires.

**Results:**

Out of a total of 770 household heads interviewed, 323 (42 %) and 447 (58 %) were males and females, respectively. The average family size in the surveyed households was 5.25 (±0.70 SE, range 1–12). The majority (81.1 %) of the households owned at least one LLIN. The average numbers of LLINs being used and sleeping places in the households were 1.61 (0.04 SE, range 0–4) and 2.27(0.03 SE, range 1–6), respectively. Most of the respondents (*n* = 687; 89.2 %) believed LLINs prevent malaria by killing or acting as physical barriers against mosquitoes. About 21 and 14 % of the respondents considered children under five years and pregnant women as priority groups for sleeping under LLINs. Households use LLINs consistently throughout the year (86.4 %) and tuck nets into bedding materials while sleeping (90.1 %). Physical inspection of fabric integrity of sampled LLINs revealed holes ranging from size 1 (0.5–2 cm) to size 4 (> 25 cm) mostly on lower (“right/left”) surfaces. Moreover, most surfaces of sampled LLINs caused 100 % knockdown and mortality on insectary colonies of *Anopheles arabiensis*.

**Conclusions:**

The overall knowledge, attitude and practice about LLINs were satisfactory in the study areas. However, the family size net ratio must be narrowed and attention needs to be given to malaria vulnerable groups (children < 5 years of age and pregnant women). Continuous monitoring and evaluation of use and durability (fabric integrity and insecticidal activity) of LLINs should be conducted to avoid misuse and associated attrition of nets before the intended period.

## Background

Malaria accounted for 198 million cases with the heaviest burden in the WHO African region where 78 % of malaria related deaths occurred in children aged less than five years. Between 2005 and 2013 the region received significant amount of funds to curb malaria with promising achievements [1].

Since the 1950’s Ethiopia has been struggling to combat malaria with chemotherapy and vector control. In its recent strategic plan on malaria [2], the country targets by 2020 (1) to achieve not more than one confirmed malaria death per 100,000 population at risk; (2) to reduce malaria cases by 75 % from the baseline in 2013 and (3) to eliminate the disease from selected low transmission areas. Integrated community health approach involving early diagnosis, effective treatment and selective vector control are among the key strategies for achieving the stated targets. In the past few years, the country has achieved substantial reduction of the burden of malaria from populations at risk [2, 3].

Despite the above promising outcomes, Ethiopia still needs to do much for malaria elimination [4]. Among others, the unstable seasonal malaria coupled with emergence of drug resistant *Plasmodium* and insecticide resistance in *Anopheles* mosquitoes are bottlenecks for malaria prevention and control in Ethiopia [5–10].

The use of LLINs are among the major malaria vector control strategies in Ethiopia [3]. By 2020 the country foresees 100 % ownership of LLINs by households in target areas with an allocation rate of at least one LLIN per two persons in a household. The plan for 2020 also involves achieving over 80 % use by all age and biological groups [2].

However, the success of insecticidal net use for malaria prevention depends on perception of the target human population and behaviour of the local malaria vector mosquitoes [11]. Lack of knowledge about priority groups to sleep under LLINs, perceiving insecticidal nets to have negative side effects and inappropriate frequency of use are among factors that hamper the role of insecticidal nets in preventing malaria in Ethiopia [12–16].

Wolaita is one of the thirteen administrative zones in the Southern Nations Nationalities and Peoples Region (SNNPR) in Ethiopia. All the 12 administrative districts of the zone are malarious with recorded cases of malaria throughout the year. Malaria is ranked first among the top ten diseases in nearly all of the districts [17]. The use of long-lasting insecticidal nets is one of the major malaria vector control strategies in the zone. Out of 366,649 malaria exposed households in the zone, 190,599 (52 %) have received at least one LLIN in the years 2012 and 2013 [17]. Like in other malarious areas of Ethiopia, health extension workers (HEWs) are meant to verify proper utilization of malaria prevention methods including LLINs. However, the monitoring and evaluation that is in place about use of LLINs was not adequate [17]. There were also no published data on the knowledge, attitudes and practice (KAP) of households about use of LLINs. Moreover, there was no information about fabric integrity and insecticidal activity of LLINs under operation. Therefore, the objectives of this study were to (i) document households’ perception (knowledge, attitude and practice) about use of LLINs; and (ii) assess fabric integrity and insecticidal activities of LLINs under operation.

## Methods

### Study area and design

Wolaita zone has an area of 4471.3 km^2^. It is located 350 km south of Addis Ababa, the capital of the country. The total population of the zone is estimated to be 1,527,908. It is one of the most densely populated zones in Ethiopia with an average of 290 people/km^2^. The livelihood of the population depends on subsistence farming [18, 19].

**Fig. 1:**
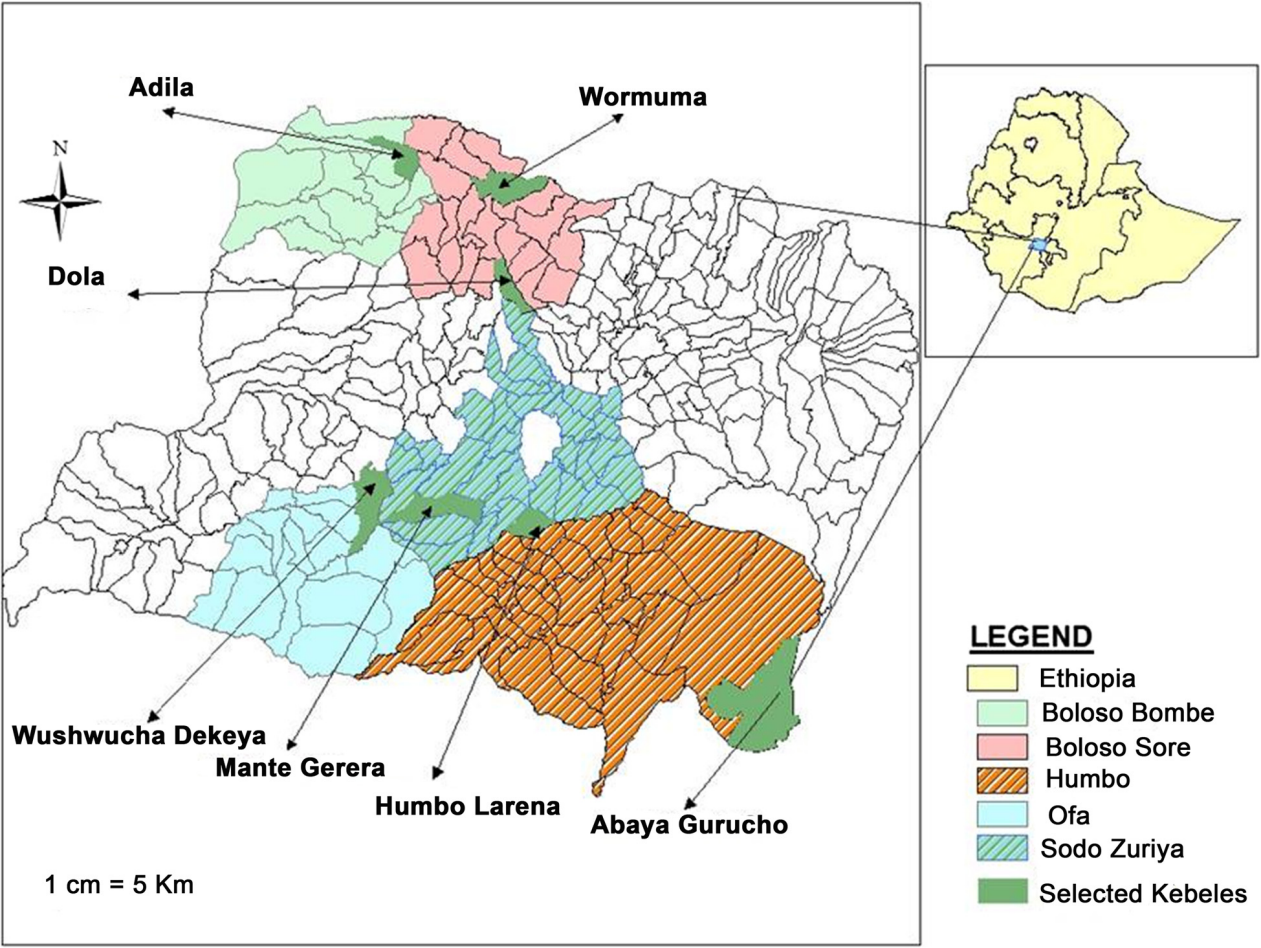
Map of the study area

A total of 7 malarious kebeles (the smallest administrative unit of Ethiopia) that were distributed in 5 malarious districts were purposefully selected for the study in consultation with zonal and district health bureaus. The kebeles selected for the study were: Humbo Larena and Mante Gerera from Sodo Zuriya district, Wushwucha Dekeya from Ofa district, Abaya Gurucho from Humbo district, Dola and Wormuma from Boloso Sore district and Adila from Boloso Bombe district (Fig. 1). Two kebeles were selected from each of Sodo Zuriya and Boloso Sore districts because of their proximity to Wolaita Sodo University and the zonal town, Wolaita Sodo. Each of the kebeles possessed a health post and a cluster health centre.

### Sample size determination

The household sample size was calculated using the standard formula for estimating a single proportion, *n* = Z^2^ P (1-P)/d^2^. Where n is estimated sample size, Z is critical value (1.96) at 95 % confidence level, P is an expected prevalence (50 %) and d is precision or margin of error (3.5 %) [20]. Since there were no previously established data about households’ perception of LLINs utilization in the study area, perception rate of 50 % was assumed and hence, the sample size calculated was 784.

### Sampling technique and interview

We assumed a comparable number of households in each of the seven kebeles and took an equal number of samples, 110 from each. The list of households in the kebeles’ administration offices was used as a sampling frame. Households were randomly selected by considering every other in the sampling frame. Household heads, spouses or their representatives aged ≥ 18 years were considered for the interview. A semi-structured interview questionnaire for monitoring durability of LLINs under operational conditions [21] was adapted to the study. The questionnaire was composed of variables such as characteristics of households, demographic profile of the respondents and household heads’ perception (knowledge, attitude and practice) about utilization of LLINs. Interviewers were trained prior to data collection and information was collected using the local Wolaita language. The interviews were conducted between March and May 2014.

### Inspection of fabric integrity of LLINs under operation

Fabric integrity of a total of 116 sample Permanet 2.0® LLINs in use were assessed by counting the number of holes of different sizes on the roof, upper surface, lower surface and seams. When present, holes were counted and categorized into four: (1) holes that did not allow a thumb to pass through [0.5–2 cm in diameter]; (2) holes that did not allow a closed fist to pass through [2–10 cm in diameter]; (3) holes larger than a fist but smaller than a head [10–25 cm in diameter] and (4) holes larger than a head [> 25 cm in diameter]. The sample nets were also inspected for repairs on all of the surfaces. When present, repairs were counted and categorized into 3: (1) stitch, (2) knot and (3) patch [21].

### Testing insecticidal activity of LLINs under operation

A total of six, 2 year-old (year of net receipt was confirmed by the heads of household) Permanet 2.0® LLINs under operation were collected from households for evaluating their insecticidal activity. The sample nets were packaged in separate clean envelopes, labelled and transported to the Aklilu Lemma Institute of Pathobiology (ALIPB), Addis Ababa University. Pieces of net samples (40 × 40 cm) were cut from upper, “left/right 1”, “left/right 2” “head/feet 1” and “/head/feet 2” surfaces of the sample nets.

Two to 3 day-old, non blood-fed female *Anopheles arabiensis* were exposed for three minutes on a net piece from each surface according to standard WHO cone bioassay procedures [21]. Knock-down and mortality data were recorded after 60 min of exposure and 24 h of holding period, respectively. Control mortality was checked by exposing mosquitoes to untreated nets (SAFI NET produced by A to Z manufacturers). *Anopheles arabiensis* used for the test was an insectary colony at the ALIPB since 2001. The test mosquitoes were susceptible to pyrethroids.

### Data analysis

Data were coded, cleaned and summarized using a Microsoft Excel spread sheet. Chi-square (χ^2^) tests were used to verify possible associations between demographic profiles of the household heads and their response to KAP questionnaires using SPSS version 10 (SPSS, Inc. USA). Probability values were considered statistically significant when the calculated *P-value* was equal to or less than 0.05. One way ANOVA was used to compare the mean number LLINs received during the previous distribution and nets under use at the time of interview among the villages.

### Ethical issues

The study was reviewed and approved by the Ethical Clearance Committee of the College of Natural and Computational Sciences, Wolaita Sodo University, Ethiopia. Permissions were obtained from zonal and district health bureaus to carry out the study. Verbal consent was obtained prior to interviewing household heads and assessing sample LLINs under use for fabric integrity. Moreover, replacement LLINs of same brand was given to households as replacement for LLINs taken for insecticidal activity test.

## Results

### Profile of the respondents

Out of 784 study subjects, 770 (98.2 %) and 14 (1.8 %) attended and missed the interview schedule, respectively. Out of 770 household heads who responded, 323 (42 %) and 447 (58 %) were males and females, respectively. The average age of the respondents was 37 years (±0.41 SE, range 18–90). The average family size was 5.25 (±0.70 SE, range 1–12). The majority of the respondents (44.2 %) had no formal education and most of them (83.4 %) were engaged in farming as a fulltime occupation (Table 1).

**Table 1:** Overview of respondents’ profile

Variables (*n* = 770)	Alternatives	Frequency
		No. (%)
Sex	Male	323 (42)
	Female	447 (58)
Marital status	Unmarried	54 (7.0)
	Married	716 (93)
Education	No. formal education	340 (44.2)
	Attended religious school	7 (0.9)
	Primary school	316 (41)
	Secondary school	86 (11.2)
	Tertiary (higher education)	21 (2.7)
Occupation	Farmer	642 (83.4)
	Governmental or NGO employee	15 (1.9)
	Small scale trader	100 (13)
	Daily laborer	13 (1.7)
Religion	Christian	770 (100)
	Other	0

### Characteristics of the surveyed households

The average number of sleeping places used by households in the previous night including temporary places outdoors was 2.27 (0.03 SE, range 1–6). The average number of LLINs received during the previous distribution (replacement included) and those under use by the households at the time of interview were 2.09 (0.03 SE, 1–4) and 1.61 (0.04 SE, range 0–4), respectively. The mean number of nets received during previous distribution and nets under use at the time of interview were significantly different among the kebeles (*P* < 0.001) . Most of the households lack electricity, consequently oil lamp was the most frequently reported source of open flame for lighting the houses. The floors of the majority of the houses were made of soil; and a considerable number of households use mat or leather on floor as a bedding material (Table 2).

**Table 2:** General characteristics of the surveyed households

Variables (*n* = 770)	Alternatives	Frequency
		No. (%)
Electricity	Yes	74 (9.6)
	No	696 (90.4)
Presence of radio or Television	Yes	203 (26.4)
	No	567 (73.6)
Floor	Soil	664 (86.2)
	Wooden or bamboo	106 (13.8)
Wall of the house	Wooden frame plastered with mud	721 (93.6)
	Wood frame covered with thatch	49 (6.4)
Roof	Wood frame covered with thatch	289 (37.5)
	Corrugated iron	481 (62.5)
Separate kitchen	Yes	350 (45.5)
	No	420 (54.5)
Open flame used in the house	Wood fire	151 (19.6)
	Charcoal fire	30 (3.9)
	Wax candle	6 (0.8)
	Oil lamp	583 (75.7)
Separate house for livestock	Yes	187 (24.3)
	No	583 (75.7)
Bedding material used	Mat or leather on floor	294 (38.18)
	Foam or grass mattress	92 (11.95)
	Wooden bed (finished)	192 (24.94)
	Wooden bed (stick frame)	192 (24.94)

### Knowledge and attitude of households about utilization of LLINs

Knowledge and attitudes of the household heads on utilization of LLINs is summarized in Table 3. Seven hundred and forty one (96.2 %) of the respondents believed that sleeping under LLINs prevent malaria. Knowledge of the respondents on the role of LLINs in preventing malaria was affected by kebeles (*χ*^*2*^ = 35.832; *P* < 0.001). The majority of the respondents reported LLINs to prevent malaria by killing or acting as physical barriers against mosquitoes. Most of the respondents with this understanding have attended at least religious school (*χ*^*2*^ = 34.824; *P* = 0.004). Moreover, 21.2 and 14.2 % of the respondents claimed children < 5 years old and pregnant women as priority groups to sleep under LLINs. However, 29.9 % of the respondents reported that there was no priority group to sleep under LLINs in their households.

**Table 3:** Knowledge and attitude of household heads on utilization of LLINs

Variables (*n* = 770)	No. (%)	Associations			
Responses		Age	Sex	Education	Village
		(*χ* ^*2*^, *P*-value)	(*χ* ^*2*^, *P*-value)	(*χ* ^*2*^, *P*-value)	(*χ* ^*2*^, *P*-value)
Sleeping under LLINs prevent malaria					
Yes	741 (96.2)	(23.640, 0.071)	(2.164, 0.141)	(2.177, 0.703)	(35.832, < 0.0001)
No	29 (3.8)				
LLINs prevent malaria by way of					
Avoiding or killing mosquitoes	687 (89.2)	(76.583, 0.073)	(7.113, 0.130)	(34.824, .004)	(4.100E^2^, < 0.0001)
Poisoning mosquitoes	16 (2.1)				
Avoiding dirt	8 (1.0)				
Avoiding or killing flies, fleas or bedbugs	15 (1.9)				
Avoiding cold	44 (5.7)				
Priority groups to sleep under LLINs					
Adults >15 years old	175 (22.7)	(74.124, .104)	(20.878, < 0.0001)	(39.275, .001)	(2.476E^2^, < 0.0001)
Children years 5–15 old	93 (12.1)				
Children < 5 years old	163 (21.2)				
Pregnant women	109 (14.2)				
No priority group	230 (29.9)				
Utilization of LLINs					
Very easy	207 (26.9)	(70.906, 0.008)	(59.956, < 0.0001)	(49.552, < 0.0001)	(5.570E^2^, < 0.0001)
Easy	381 (49.5)				
A bit difficult	53 (6.9)				
Too difficult	129 (16.8)				
Willingness to buy LLINs					
Yes	207 (26.9)	(33.011, 0.005)	(26.357, < 0.0001)	(16.917, 0.002)	(1.576E^2^, < 0.0001)
No	563 (73.1)				
Experiencing side effects of LLINs					
Yes	124 (16.1)	(21.219, 0.130)	(28.743, < 0.0001)	(5.830, 0.212)	(80.303, < 0.0001)
No	646 (83.9)				

On the other hand, 26.9 % of the respondents were willing to buy LLINs if provided with an affordable price. The attitudes of the respondents towards buying LLINs for their households was significantly different between males and females (*χ*^*2*^ = 26.357; *P* < 0.001).

### LLIN utilization of households

The majority (81.1 %) of households owned at least one LLIN at the time of interview. Household heads with primary education had higher net ownership (*χ*^*2*^ = 13.392; *P* = 0.010). Moreover, net ownership was higher in Mante Gerera kebele followed by Abaya Gurucho and Humbo Larena (*χ*^*2*^ = 2.691E^2^; *P < *0.001) (Table 4). Most of the respondents claimed that their household members sleep under LLIN all year round. However, sex (*χ*^*2*^ = 10.853; P = 0.013) and site (*χ*^*2*^ = 93.691; *P* < 0.001) had effect on season of sleeping under LLINs. The majority of the household heads reported that they did not use LLINs for other purposes. However, this was affected by sex (*χ*^*2*^ = 26.031; *P* < 0.001), education (*χ*^*2*^ = 13.655; *P* = 0.008) and site (*χ*^*2*^ = 1.398E^2^; *P* < 0.001). About 53 % of the households hang LLINs above their sleeping location every night. The sex of the respondents (*χ*^*2*^ = 16.733; *P* < 0.001) and site (*χ*^*2*^ = 115.12; *P* < 0.001) had significant effects on the timing of hanging LLINs. More household heads with primary education tucked the lower edge of their LLIN(s) into their bedding materials than household heads with no formal education (*χ*^*2*^ = 16.798; *P* = 0.002). Four hundred and fifty two (58.7 %) of the interviewees reported that they scrub their LLIN(s) on hard surfaces while washing. More female household heads claimed to scrub the nets on hard surfaces than males (*χ*^*2*^ = 6.066; *P* = 0.014). Moreover, the highest proportion of households in Mante Gerera kebele reported to scrub the LLINs on hard surfaces while washing (*χ*^*2*^ = 68.163; *P* < 0.001). Furthermore, 53 % of the household heads reported that they did not squeeze LLINs after washing. A higher proportion of households that did not squeeze nets after washing were observed in Adila kebele (*χ*^*2*^ = 28.882; *P* < 0.001). Regarding drying of LLINs after washing, “sun drying”, “under shade drying” and “indoor drying” accounted for 42.2, 53 and 2.1 %, respectively. The proportion of female household heads that claimed to dry the LLINs in the sun after washing were higher than that of the males (*χ*^*2*^ = 25.300; *P* < 0.001). Moreover, the highest proportion of households in Mante Gerera kebele practice sun drying nets outdoors (*χ*^*2*^ = 1.809E^2^; *P* < 0.001)*.*

**Table 4:** Practice of household heads about ownership and utilization of LLINs

Variables (*n* = 770)	No. (%)	Associations			
Responses		Age	Sex	Education	Site
		(*χ* ^*2*^, *P*-value)	(*χ* ^*2*^, *P*-value)	(*χ* ^*2*^, *P*-value)	(*χ* ^*2*^, *P*-value)
Receipt of LLIN(s) during previous distribution					
Yes	677 (87.9)	(18.047, 0.260)	(36.580, < 0.0001)	(8.413, 0. 078)	(2.691E^2^, < 0.0001)
No	93 (12.1)				
Training about utilization of LLINs					
Yes	722 (93.8)	(30.736, 0.010)	(2.406, 0.121)	(3.953, 0.412)	(18.797, 0.005)
No	48 (6.2)				
Possession of LLINs at the time of interview					
Yes	630 (81.8)	(12.823, 0.616)	(0.498, 0.480)	(13.392, 0.010)	(1.303E^2^, < 0.0001)
No	140 (18.2)				
Frequent sleeping under LLIN					
Yes	593 (77.0)	(18.920, 0.217)	(12.351, < 0.0001)	(20.560, < 0.0001)	(1.740E^2^, < 0.0001)
No	177 (23.0)				
Season of frequent sleeping under LLINs					
All year round	665 (86.4)	(35.970, 0.830)	(10.853, 0.013)	(17.635, 0.127)	(93.691, < 0.0001)
Rainy season only	80 (10.4)				
Dry season only	6 (0.8)				
Do not know	19 (2.5)				
Utilizing LLINs for non intended purposes					
Yes	201 (26.1)	(13.516, 0.563)	(26.031, < 0.0001)	(13.655, 0.008)	(1.398E^2^, < 0.0001)
No	569 (73.9)				
Time of hanging LLIN					
Every night	409 (53.1)	(41.625, 0.077)	(16.733, < 0.0001)	(15.305, 0.053)	(1.151E^2^, < 0.0001)
Remains hanged day and night	351 (45.6)				
When the weather is cold	10 (1.3)				
Tucking the net into the sleeping material					
Yes	694 (90.1)	(20.945, 0.139)	(2.838, 0.092)	(16.798, 0.002)	(45.665, < 0.0001)
No	76 (9.9)				
Training on how to wash LLINs					
Yes	563 (73.1)	(17.546, 0.287)	(18.106, < 0.0001)	(2.026, 0.731)	(69.177, < 0.0001)
No	207 (26.9)				
Washing the LLIN(s)					
Yes	694 (90.1)	(33.285, 0.004)	(2.245, 0.134)	(1.257, 0.869)	(38.512, < 0.0001)
No	76 (9.9)				
Frequency of washing LLINs					
Every week	85 (11.0)	(37.808, 0.155)	(2.650, 0.266)	(18.007, 0.021)	(1.237E^2^, < 0.0001)
Every month	221 (28.7)				
Every three-six months	464 (60.3)				
Type of soap used for washing the LLNs					
None	21 (2.7)	(50.334, 0.011)	(7.090, 0.029)	(22.848, 0.004)	(1.483E^2^, < 0.0001)
Bar soap from local market	638 (82.9)				
Powder soap from local market	111 (14.4)				
Time interval of soaking LLINs while washing					
None	332 (43.1)	(44.255, 0.045)	(32.448, < 0.0001)	(9.446, 0.306)	(2.704E^2^, < 0.0001)
For < 1 h	426 (55.3)				
For > 1 h	12 (1.6)				
Scrubbing the net on hard surfaces while washing?					
Yes	452 (58.7)	(21.163, 0.132)	(6.066, 0.014)	(3.970, 0.410)	(68.163, < 0.0001)
No	318 (41.3)				
Squeezing the net after washing					
Yes	362 (47.0)	(12.603, 0.633)	(1.792, 0.181)	(2.443, 0.655)	(28.882, < 0.0001)
No	408 (53.0)				
Place of drying LLINs after washing					
Outdoor (sun drying)	340 (44.2)	(24.416, 0.753)	(25.300, < 0.0001)	(5.678, 0.683)	(1.809E^2^, < 0.0001)
Outdoor (dry under shade)	414 (53.8)				
Indoor	16 (2.1)				

### Fabric integrity of LLINs

The average number of holes of different sizes and repairs identified in the LLINs inspected are summarized in Tables 5 and 6, respectively. Higher mean number of holes of all categories occurred on the lower surfaces of the nets. On the other hand, stitching was the most frequently observed method of repairing nets.

**Table 5:** Holes of different sizes on under use LLINs

	Type of holes	Surfaces of LLIN inspected			
		Roof	Upper	Lower	Seams
Size 1	Mean	1.3017	2.8793	4.5000	0.91
	Standard Error	0.14907	0.96794	1.73890	0.126
	Minimum	0	0	0	0
	Maximum	7.00	101.00	200.00	6
Size 2	Mean	0.99	1.66	2.74	0.79
	Standard Error	0.134	0.306	0.383	0.118
	Minimum	0	0	0	0
	Maximum	6	27	26	6
Size 3	Mean	0.73	0.84	1.29	0.76
	Standard Error	0.100	0.112	0.128	0.109
	Minimum	0	0	0	0
	Maximum	5	6	8	5
Size 4	Mean	0.62	0.86	0.99	0.72
	Standard Error	0.097	0.110	0.132	0.120
	Minimum	0	0	0	0
	Maximum	4	5	5	5

**Table 6:** Distribution of repairs on LLINs under use

Type of repair	Mean ± SE	Minimum	Maximum
Stitch	1.4 ± 0.2	0	9
Knot	0.6 ± 0.09	0	4
Patch	0.7 ± 0.11	0	4

### Insecticidal activity of LLINs under operation

**Fig. 2:**
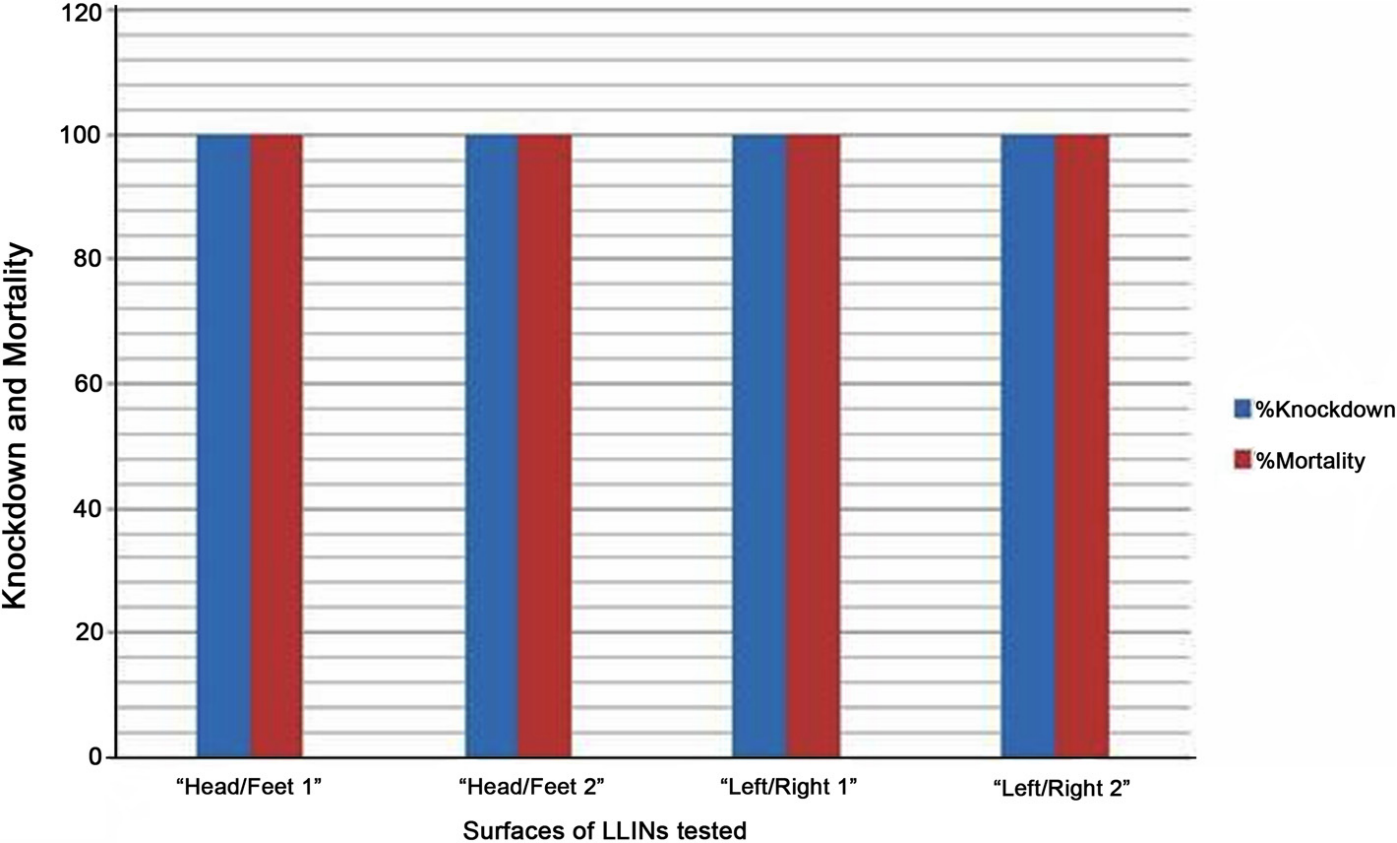
Insecticidal activities of surfaces of LLINs from Mante Gerera kebele

**Fig. 3:**
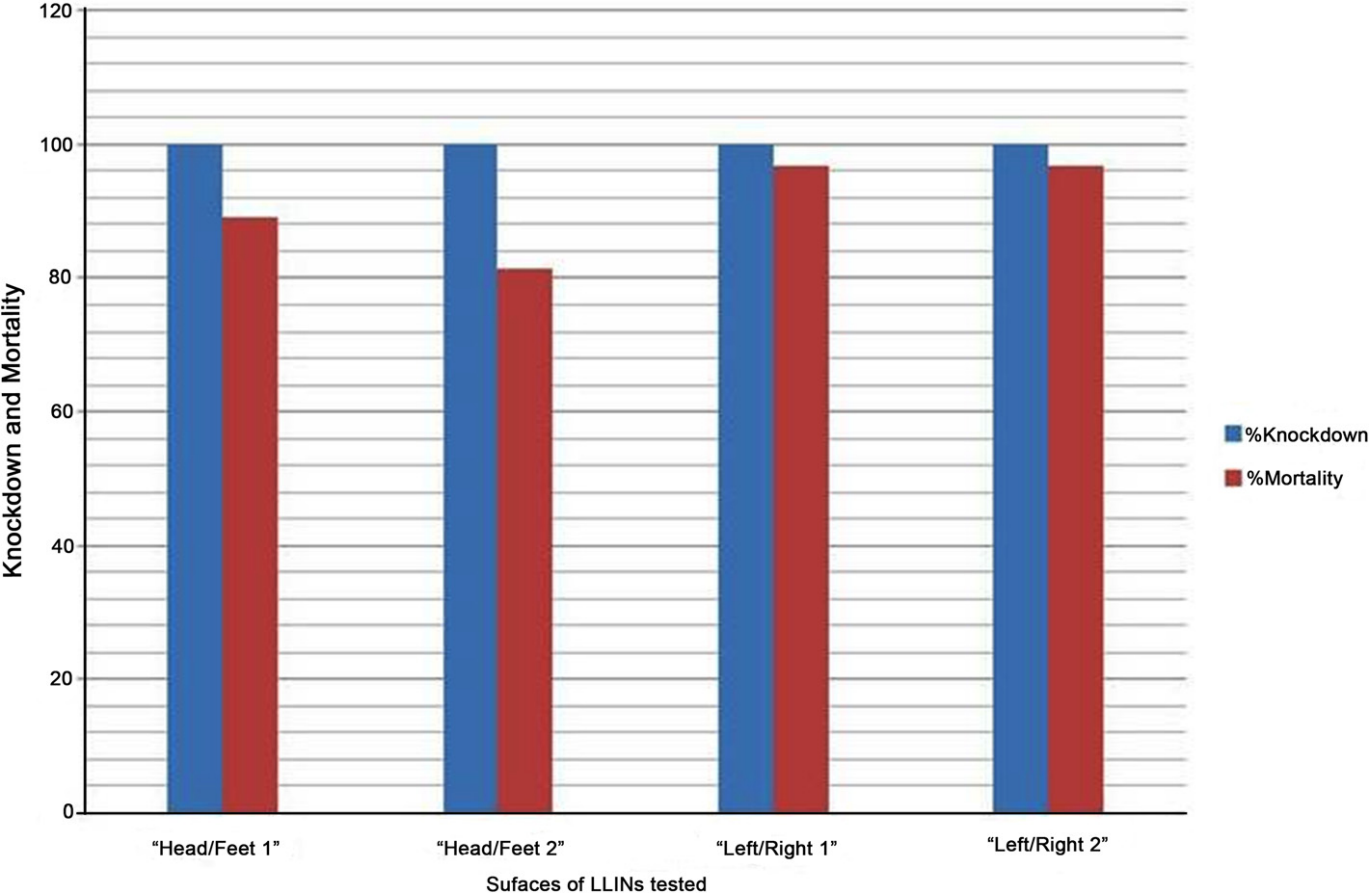
Insecticidal activities of surfaces of LLINs from Wushwucha Dekeya kebele

All test surfaces of LLINs from Mante Gerera kebele showed 100 % knockdown and 100 % mortality of *An. arabiensis* after 60 min exposures and 24 h of holding period, respectively (Fig. 2). On the other hand, test surfaces of LLINs from Wushwucha Dekeya kebele resulted in 100 % knockdown and mortalities ranging from 81 to 97 % on *An. arabiensis* (Fig. 3).

## Discussion

This study is perhaps the first to document the perception about utilization of LLINs from households and assess fabric integrity and insecticidal activity of LLINs under operation in rural kebeles of Wolaita Zone.

In general, there was discrepancy between the average family size, 5.25 (± 0.70 SE, range 1–12), and the average number of functional LLINs, 1.61 (0.04 SE, range 0–4), owned by the households at the time of the survey. This contradicts the WHO target of allocating one LLIN for every two people in the household [22]. The disproportion also existed between the average numbers of nets, 1.61 (0.04 SE, range 0–4), and average number of sleeping places (including temporary places outdoor), which was 2.27 (0.03 SE, range 1–6).

About eighty-nine percent of the respondents in this study believed LLINs prevent malaria by way of killing or acting as a physical barrier against mosquitoes. This is very close to the awareness of households in Kola Tembien district, North Ethiopia [16], Wonago district, Southern Ethiopia [13] and Oromia and Amhara regions of Ethiopia [23]. Moreover, this study revealed better awareness of households about how LLINs prevent malaria than the report from malaria prone regions of Ethiopia [14]. Only 21 and 14 % of the respondents prioritized children below five years of age and pregnant women, for sleeping under LLINs. On the contrary, studies elsewhere in Ethiopia reported children below five years of age as the highest priority group for sleeping under LLINs [14, 16, 24]. Moreover, prioritizing pregnant women for sleeping under LLINs was higher in this study compared to the report by Animut et al. [14] and Tomas et al*.* [16] but lower compared to the report by Kassahun et al. [24]. These findings imply varying levels of awareness of communities about vulnerability of children less than five years of age and pregnant women to malaria in different parts of Ethiopia.

More than 70 % of the respondents in this study were not willing to buy nets even if they are provided with affordable prices. This is in agreement with a study in Arbaminch and its suburbs, in southern Ethiopia where about 62 % of the study subjects preferred free distribution of nets [25]. However, in this study the perception of household heads to buy nets varied with gender. Generally, community preference for free distribution of bed nets may discourage selling of nets by households. On the other hand, free distribution of LLINs enhanced utilization elsewhere [26, 27].

The majority (81.1 %) of the households own at least one LLIN and this is comparable with the report from Afar (86.1 %), [24] and Kafta-Humera (85.5 %), [28] in Ethiopia. However, a higher proportion (97 %) of households owned at least one LLIN in Jabi Tehnan district of north-western Ethiopia [29]. In the present study, ownership of at least one LLIN varied with education and kebele. In the present study, a higher proportion of female household heads claimed to sleep under LLINs consistently throughout the year than the males. The obvious reason for this might be females are the most accessible in households and might have received better health education about malaria prevention than males.

About 26 % of the interviewees used LLINs for unintended purposes. This was further substantiated by our observation of households misusing LLINs as bed sheets and covers for bedding materials attempting to kill blood-sucking insects such as fleas and bed bugs. Moreover, during the survey it was common to see households using intact nets for packaging and displaying bananas in the local markets. Some households were also observed to use LLINs for ripening bananas. Such misuses of LLINs were reported elsewhere in the country [30]. Most of the respondents in this study were engaged in subsistence farming and were observed using LLINs for covering harvested crops before threshing.

The majority of households tuck the lower edge of LLINs into bedding materials while sleeping and this is inconsistent with the report from Afar [25].

In this study the “right” or “left” long sides of the LLINs were considered as “lower surfaces”. Holes of all the four size categories were observed on these surfaces. These surfaces of the LLINs are very close to floors of houses and exposed to various household factors that potentially damage fabric integrity of the nets. Poor hanging was observed to be one of the factors that bring nets in contact with floors of houses where the ground forms the sleeping places. Moreover, lack of electricity and using open flame in the houses including in rooms where LLINs are hung might have caused the holes. On the other hand, co-existence of domestic livestock with humans in the households might have contributed to the wear-and-tear on the nets. Household heads in the study areas also claimed that rodents were among the causes for net damage. The damage of insecticidal nets by rodents has been reviewed [31].

Furthermore, the insecticidal activities of two year-old Permanet 2.0® long-lasting insecticidal nets were highly effective in knocking down and killing laboratory colonies of *An. arabiensis*. This implies that the nets possessed insecticidal activities despite the prevailing misuse, wear and tear as well as inappropriate washing practices. However, in order to come up with strong recommendations about insecticidal activities of LLINs under operation, it is crucial to carry out similar tests against field collected local malaria vector mosquitoes.

## Conclusions

The overall perception of households about utilization of LLINs was satisfactory. However, there was imbalance between family size and number of LLINs owned. Most of the respondents did not identify priority groups for sleeping under LLINs in their households. Physical inspection of LLINs revealed holes ranging from size 1 (0.5–2 cm) to size 4 (> 25 cm). Moreover, old (at most two years) Permanet 2.0® LLINs under operation were highly effective in knocking down and killing insectary colonies of *An. arabiensis*. However, testing insecticidal activities of LLINs under operation against field populations of malaria vector mosquitoes would provide substantial evidence about insecticidal activity of the nets. Wide ranges of misuse and factors that may result in wear and tear on LLINs have been observed in the study area. Therefore, well-tailored awareness creation and continuous monitoring and evaluation needs to be in place to avoid misuse and associated attrition of nets before the intended period.
